# A Narrative and Critical Review of Randomized-Controlled Clinical Trials on Patent Foramen Ovale Closure for Reducing the Risk of Stroke Recurrence

**DOI:** 10.3389/fneur.2020.00434

**Published:** 2020-06-04

**Authors:** Apostolos Safouris, Odysseas Kargiotis, Klearchos Psychogios, Pericles Kalyvas, Ignatios Ikonomidis, Maria Drakopoulou, Konstantinos Toutouzas, Georgios Tsivgoulis

**Affiliations:** ^1^Stroke Unit, Metropolitan Hospital, Pireus, Greece; ^2^Second Department of Neurology, School of Medicine, Attikon University Hospital, National and Kapodistrian University of Athens, Athens, Greece; ^3^Cardiology Department, Metropolitan Hospital, Pireus, Greece; ^4^Department of Echocardiography and Laboratory of Preventive Cardiology, Second Cardiology Department, Attikon Hospital, National and Kapodistrian University of Athens, Athens, Greece; ^5^First Department of Cardiology, Athens School of Medicine, Hippokration Hospital, Athens, Greece; ^6^First Department of Cardiology, Medical School of Athens University, Hippokration Hospital, Athens, Greece; ^7^Department of Neurology, University of Tennessee Health Science Center, Memphis, TN, United States

**Keywords:** patent foramen oval, PFO closure, stroke, cryptogenic, randomized controlled (clinical) trial

## Abstract

Patent foramen ovale (PFO) is a common cardiac anatomic variant that has been increasingly found in young (<60 years) cryptogenic stroke patients. Despite initial neutral randomized-controlled clinical trials (RCTs), there have been four recent RCTs providing consistent data in favor of the efficacy and safety of PFO closure compared to medical therapy for secondary stroke prevention. However, taking into consideration the high prevalence of PFO, the low risk of stroke recurrence under medical treatment and the uncommon yet severe adverse events of the intervention, patient selection is crucial for attaining meaningful clinical benefits. Thorough workup to exclude alternative causes of stroke and identification of high-risk PFOs through clinical, neuroimaging and echocardiographic criteria are essential. Cost effectiveness of the procedure cannot be proven for the time being, since there are no robust data on clinical outcome after PFO-associated stroke but only limited anecdotal data suggesting low risk for long-term disability.

## Introduction

Foramen ovale is a component of the fetal cardiovascular circulation that during postnatal life closes in ≈70% of subjects, whereas in the remaining 30%, remains patent as a tunnel and converts into a “flap-like” valve that may open every time the right atrial pressure overcomes the left one. Patent foramen ovale (PFO) is therefore a normal variant of the atrial septum rather than a congenital heart defect. PFO has been associated with cryptogenic ischemic stroke especially in younger patients (<60 years) after several seminal epidemiological studies in the 90's have shown a statistically significant association (1–4). Estimates on prevalence vary considerably depending on the population and the diagnostic method used (5). PFO is detected on transesophageal echocardiography in 1 out of 4–5 individuals whereas among younger patients with cryptogenic ischemic stroke, PFOs is present in more than 50% of cases. Transthoracic echocardiography bubble study is commonly used for the diagnosis of PFO in patients with cryptogenic stroke. Transcranial Doppler (TCD) is a bedside, non-invasive investigation of the cerebral blood flow that has also been evaluated as a potential screening tool for the detection of a right-to-left shunt (RLS) (6). TCD showed greater sensitivity and overall diagnostic accuracy but lower specificity compared to transthoracic echocardiography for the detection of PFO in cryptogenic stroke patients in a meta-analysis of prospective observational studies (7). Transesophageal echocardiography (TEE) bubble study is currently considered the gold standard for PFO investigation. A meta-analysis of prospective studies determined that TEE bubble study has a sensitivity of 89% and specificity of 91% when compared to confirmation by autopsy, surgery, and/or right heart catheterization. False negative and false positive results may occur due to technical limitations including patient intolerance for the probe, inadequate Valsalva maneuver during sedation and operator experience (8, 9). TCD is more sensitive (sensitivity: 95–98%) compared with TEE (sensitivity: 80–100%) but carries a lower specificity, diagnosing not PFO *per se* but only RLS; it also fails to provide any information about other potential cardiac and aortic embolic sources (10, 11).

PFO width ranges widely in adults from 1 to 19 mm (mean 4.9 mm). Depending on its size, which may be echocardiographically evaluated by measuring the maximum opening between septum primum and septum secundum in the left atrium, PFO can be classified as large ≥4 mm, medium 2–3.9 mm and small <2 mm. Certain PFO characteristics as described by TEE may increase the association with cryptogenic stroke (Table 1).

**Table 1:** PFO anatomical features associated with high risk for stroke.

1	Long tunnel (>10 mm)
2	Atrial septal aneurysm (>10 mm from the midline of the atrial septum)
3	Thick septum secundum (>10 mm)
4	Multiple orifices in the left atrium
5	Eustachian valve or Chiari network

**Figure 1:**
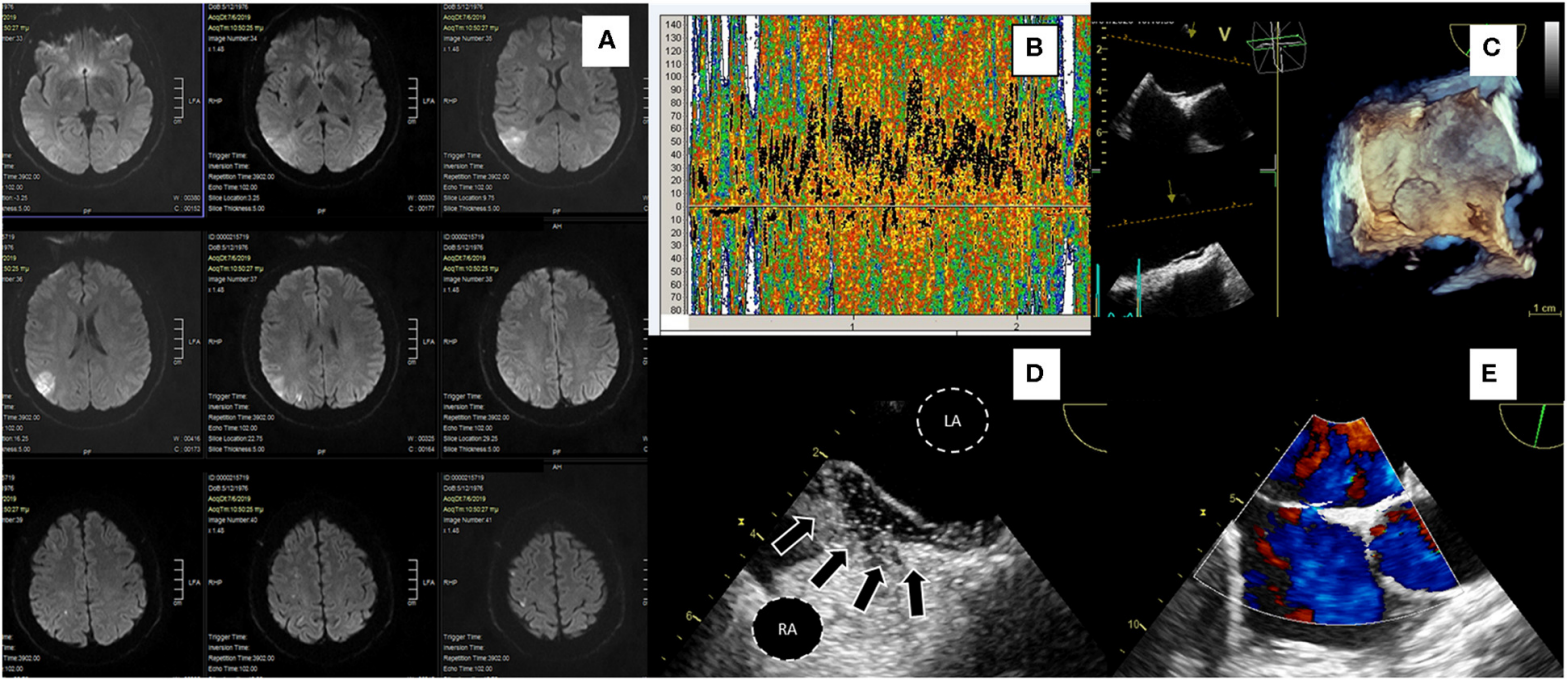
A 43-yr-old hypertensive male patient presented with a sudden-onset 15-min episode of left hemiparesis. Diffusion-weighted MRI **(A)** showed an acute ischemic right parietal cortical lesion with multiple concomitant ischemic lesions, all in the distribution of right middle cerebral artery. Ultrasonography of extracranial and intracranial brain arteries excluded atheromatosis and TCD bubble study showed a severe (Grade III) right-to-left shunt **(B)**. TEE confirmed the presence of a large PFO without ASA; 3D TEE demonstrated septal separation **(C)**, bubble study showing microbubbles crossing through a tunnel-type PFO (arrows) (**D**, LA, left atrium; RA, right atrium). 2D TEE with color flow doppler showing a small left to right shunt through PFO during rest **(E)**. RoPE score was 7. The patient had multiple Holter ECG recordings during and after hospitalization without AF episodes or signs of atrial hyperexcitability. He was uneventfully treated with PFO closure a month after discharge.

It has been shown that increased septal mobility may mechanically direct blood flow from the inferior vena cava into the PFO, thus increasing the risk of paradoxical embolism (12). Patients with both PFO and atrial septal aneurysm (ASA) are at greater risk for recurrent stroke, compared to those with PFO only (13, 14). Distinguishing large from small PFOs might be relevant for identifying pathogenic PFOs (15), predicting stroke recurrence or selecting those patients that are more likely to benefit from closure. However, data from observational studies are controversial. In the largest study of PFO size and relation to ischemic stroke, PFO size was not a significant predictor of the index event (16). Secondary features of PFO such as the presence of a Eustachian valve, Chiari network or a long PFO tunnel were suggested to be linked to PFO-associated strokes in some retrospective studies (17, 18) but not others (19–21). A case of PFO-associated stroke is presented in Figure 1 and Supplementary Videos 1, 2. In this narrative review, we present data from randomized-controlled clinical trials (RCTs) on PFO closure for secondary stroke prevention. We have searched Pubmed for meta-analyses, observational studies, and other narrative reviews on the subject. Despite the abundance of relative literature, we have noted a paucity of robust data on clinical outcome post PFO-associated stroke. This information is requisite to correctly address cost effectiveness of PFO closure procedures, as we will discuss.

## The Association Between PFO and Stroke in Observational Studies

We have performed a systematic review of the available prospective observational studies published before April 2014, to evaluate the association of PFO with an increased risk of recurrent stroke or transient ischemic attack (TIA) in medically treated stroke patients (22). We also investigated the relationship of shunt size with the risk of recurrent cerebrovascular events. A systematic research of the literature returned 13 eligible studies to which we added the available preliminary data from an ongoing cohort study conducted by our group. Stroke patients with PFO did not show increased risk of the combined outcome of recurrent cryptogenic stroke/TIA (RR = 1.18; *P* = 0.43) or recurrent cryptogenic stroke (RR = 0.85; *P* = 0.37) in comparison with stroke patients without PFO. No substantial heterogeneity has been noted between the studies analyzed. The annual rate of recurrent stroke/TIA per 100 patient-years was 5.6% with PFO and 5.0% without PFO (*p* = 0.79), and of recurrent stroke 2 and 2.4%, respectively (*p* = 0.44). Similarly, the combined risk of recurrent cryptogenic stroke/TIA (RR = 1.33; *P* = 0.33) and the risk of recurrent cryptogenic stroke (RR = 1.34; *P* = 0.35) did not differ among patients with stroke harboring a large shunt in comparison to those with stroke and a moderate or small shunt in both the overall and subgroup analyses (separate evaluation of TCD and TEE studies); no evidence of heterogeneity has been noted. It should be noted that risk of bias was high in some of the included studies, especially concerning loss to follow-up and blinding of outcome assessment. Finally, a recent Canadian study indicated that TCD stratification of right-to-left shunt size might be more accurate for recurrent stroke risk predication in a cohort of patients with cryptogenic stroke (23). In the following years, numerous randomized-controlled clinical trials (RCTs) systematically evaluated the safety and efficacy of PFO closure for secondary stroke prevention.

## Clinical Trials of PFO Closure

Earlier RCTs have failed to demonstrate benefit of PFO closure in patients after ischemic stroke. CLOSURE I trial randomized cryptogenic stroke/TIA patients 18–60 years old to PFO closure vs. conservative treatment with various antithrombotic regimens (double antiplatelet or anticoagulation or combination of antiplatelet and anticoagulation, depending on the preferences of the treating physician) (24). No selection criteria were used regarding PFO characteristics and the Starflex device has been used for closure. Patients were followed for 2 years and the primary outcome was a composite of death or stroke/TIA. The intervention group has shown a tendency for reduced stroke recurrence, but this was not statistically significant. The umbrella-clamshell design of the Starflex has been subsequently shown to be inferior compared to double-disk devices and this could explain the neutral results of the trial (25). In the intervention arm, 3% of patients had major periprocedural vascular adverse events and in 6% of cases atrial fibrillation (AF) was detected, raising a red flag for subsequent trials.

The PC trial randomized cryptogenic stroke/TIA patients younger than 60 to medical treatment with either antithrombotic or anticoagulation, or to PFO closure with a disk device (26). The trial showed no decrease in the risk of death, stroke or TIA. However, safety concerns were mitigated since a non-significant increase of AF has been recorded in the intervention arm (2.9 vs. 1% in the medical arm, *p* = 0.6). Of the six patients that developed AF post-intervention, only one had persistent AF and none suffered stroke. There have been some cross overs from the medical to the intervention arm, either due to patient or physician preference or due to stroke, a pattern that was repeated in many of the following trials, leading to exclusion of these patients from analyses.

The RESPECT trial published its results in 2013 and the extended follow-up in 2017. Initial results were neutral, at least in the intention-to-treat cohort that showed a non-significant trend for a lower risk of stroke recurrence after PFO closure. However, the intervention proved superior in the prespecified per-protocol and as-treated analyses (27). The procedure proved safe with similar serious adverse event rates between the two treatment arms. Procedure-related or device-related serious adverse events occurred in 4.2% of the intervention arm but rates of AF or device thrombus were not significantly increased. The mixed results of the RESPECT trial failed to change treatment guidelines or clinical practice but incited hopes that the procedure may provide benefit in selected stroke patients.

A meta-analysis of the 3 trials regrouping individual data from 2,303 patients reinforced such beliefs (28). Despite the absence of significant association with the primary composite outcome in unadjusted analysis, benefit became marginally significant after covariate adjustment. Patient selection issues and inclusion of patients without cryptogenic stroke accounted for much of neutral outcomes. More importantly, PFO closure was associated with a significant reduction of the risk of stroke recurrence in both unadjusted and adjusted analyses, whereas when only the 2 trials that used disc occluder devices were included, recurrent stroke risk reduction proved clearly significant and more pronounced. The annualized rate of ischemic stroke, if treated medically, was approximately 1%; device closure decreased this rate by 50% approximately, averting 1 ischemic stroke over 2.5 years for every 67 patients treated. Rates of AF were numerically increased with device closure in the overall analysis but did not reach statistical significance. It should be noted that despite reaching statistical significance, these results could be considered marginally clinically relevant.

In contrast to these mixed results, three recent RCTs have consistently provided data in favor of recurrent stroke reduction in cryptogenic stroke patients randomized to PFO closure. Following the neutral preliminary results from the original trial period of the RESPECT trial reported in 2013, the results from the extended follow-up period (median of 6 years) were published in 2017 and showed benefit from PFO closure (29). Regarding the primary endpoint of ischemic stroke/early death with PFO closure vs. medical therapy, the intervention group showed reduced risk (HR = 0.55; *P* = 0.046). Recurrent ischemic cryptogenic stroke occurred in 10 patients in the PFO closure group as compared with 23 patients in the medical-therapy group, yielding corresponding rates of 0.32 events per 100 patient-years and 0.86 events per 100 patient-years (HR = 0.38; *P* = 0.007). These findings translate to a number needed to treat (NNT) of 42 to avoid one ischemic stroke in 5 years. The medical therapy group received antithrombotic therapy of greater intensity than the PFO closure group, including more common use of anticoagulant agents (percentage of patient-years of follow-up in which anticoagulant therapy was used, 21.6 vs. 3.3). Subgroup analysis demonstrated more pronounced benefit of PFO closure in patients harboring an atrial septal aneurysm, as well as in those with a substantial (grade 3) right-to-left shunt.

The CLOSE trial recruited only this supposedly high-risk cryptogenic stroke group: patients with a large shunt or concomitant presence of ASA (30). The trial had three treatment arms, with patients not undergoing PFO closure being randomized to either antiplatelet or anticoagulation therapy. This permitted the investigators to compare PFO closure plus antiplatelet treatment to a homogeneous medical treatment group (antiplatelet only or anticoagulation only). Definition of cryptogenic stroke was stringent, far beyond commonly used the Trial of Org 10,172 in Acute Stroke Treatment (TOAST) criteria (31). All patients underwent vascular imaging and atherosclerotic patients were excluded according to the definition of atherosclerosis in the study criteria: “Atherosclerosis: presence of stenosis ≥ 30% of an artery supplying the brain or atherosclerosis of the aortic arch (plaque ≥ 4 mm). In case of arterial occlusion in the appropriate territory, the diagnosis of atherosclerosis was adopted if the patient presented at least two cardiovascular risk factors (hypertension, diabetes, hypercholesterolemia, smoking) or a history of myocardial infarction or arterial disease of the lower limbs or an atherosclerotic stenosis (≥ 30%) of another artery supplying the brain or plaques of the aortic arch.” Even though this is an original definition that has not been validated in other studies, it reflects current knowledge on the role of non-stenotic atherosclerotic plaques in ischemic stroke (32). Maybe in part due to an extended work-up, CLOSE reported the lowest mean age (43 years) among all PFO trials and the lowest prevalence of hypercholesterolemia (14%) and hypertension (11%) among enrolled patients. The 5-year risk of stroke, according to the Kaplan–Meier probability estimate, was 4.9% in the antiplatelet-only group compared to no stroke with PFO closure, which would result in one stroke avoided at 5 years for every 20 treated patients (95% CI: 17–25). AF occurred in 4.6% of patients in the PFO closure group, mostly within 1 month after the procedure, as compared to 0.9% in the antiplatelet-only group (*P* = 0.02). Most of these cases did not necessitate lifelong anticoagulation treatment. It should be highlighted that AF occurred in 1% of the medical treatment group, meaning that despite the rigorous work-up for arterial atherosclerosis, the absence of long-term ECG monitoring may have resulted in the inclusion of a small percentage of patients with occult paroxysmal AF. Finally, the rate of recurrent disabling stroke did not differ in the two treatment groups (0 events in the PFO closure group vs. 1 event in antiplatelet treated group).

In the Gore REDUCE clinical trial, 664 patients with cryptogenic stroke (randomly assigned in a 2:1 ratio to PFO closure plus antiplatelet therapy or antiplatelet therapy alone) were followed for a median of 3.2 years (33). The investigators excluded patients that otherwise fulfilled typical criteria for cryptogenic stroke, if they had a history of prosthetic heart valve, severe native valve disease, left ventricular ejection fraction of <40%, severe ventricular wall motion abnormalities (akinesis, severe hypokinesis), prior cardiac surgery, or prior myocardial infarction, in an effort to eliminate inclusion of non-PFO-related ischemic stroke patients. They also excluded patients with uncontrolled arterial hypertension or poorly controlled diabetes mellitus at the initial screening, further limiting the inclusion of patients that would potentially not benefit from PFO closure. Only PFO presence was requisite for inclusion and atrial septal aneurysm was not systematically screened before randomization. Therefore, one out of five patients in the intervention arm proved to have ASA during closure. Notably, most included patients (4 out of 5 in both arms) had moderate (6–25 bubbles) or severe (>25 bubbles) shunt. The trial showed positive results: ischemic stroke occurred in 1.4% in the PFO closure group and in 5.4% in the antiplatelet-only group (hazard ratio, 0.23; *P* = 0.002) during a mean follow-up of 3.2 years. Exploratory analysis to evaluate heterogeneity in relation to shunt size failed to reveal a significant interaction, however, when only patients with small shunt size were examined, there was a non-significant trend (*p* = 0.26) to stroke recurrence reduction but with wide confidence intervals due to small sample size. In contrast, the effect of PFO closure was highly significant among patients with moderate-to-large shunt size. (HR: 0.18, 95% CI: 0.06–0.58; *p* < 0.001). Cross-overs were limited, and the results favored PFO closure both in the per-protocol and the as-treated cohorts. Participants were also screened for new ischemic brain lesions with a baseline and a control brain MRI 24 months later. There was no significant increase in silent brain lesions, although the total ischemic lesion burden was higher in the medical treatment arm, which is in line with the increase in clinical ischemic stroke. Atrial fibrillation or flutter occurred in significantly more patients in the PFO closure group than in the antiplatelet-only group (6.6 vs. 0.4%, *P* < 0.001). The very low incidence of newly detected AF in the medical treatment arm suggests that the exclusion criteria of the trial were efficient in uncovering cryptogenic stroke patients with covert AF. It should be noted that out of 29 patients who developed atrial fibrillation or flutter in the PFO closure group, 1 had a recurrent stroke, showing that even though PFO closure-related AF can be transient and remit in the majority of cases, it should be actively treated if it persists for days following PFO closure.

In the DEFENSE PFO trial patients with cryptogenic stroke and high-risk PFO identified using TEE criteria [atrial septal aneurysm, PFO hypermobility (phasic septal excursion into either atrium≥10 mm), PFO large anatomical size (maximum separation of the septum primum from the secundum ≥2 mm)] were randomized to either transcatheter PFO closure or medical treatment which consisted of initially double and then single antiplatelet therapy in most cases, or warfarin in 25% of patients (34). Patients with a history of myocardial infarction or unstable angina, left ventricular systolic dysfunction with ventricular wall aneurysm or akinesia were excluded. Once again, these exclusion criteria aimed to set apart patients that may have had atherosclerotic or cardioembolic etiologies of stroke even if standard work-up failed to demonstrate such a mechanism, on the grounds of medical history and echocardiographic findings. In analogy to the CLOSE trial, there has been no recurrence of stroke in patients who underwent PFO closure, suggesting that the beneficial effect of percutaneous device closure of PFO can be maximized by adding to the selection criteria PFO morphologic characteristics as depicted by TEE. The primary endpoint was a composite of stroke, vascular death, or Thrombolysis in Myocardial Infarction (TIMI)-defined major bleeding during the 2 years of follow-up, which occurred only in the medical treatment group, with a 2-year event rate of 12.9% (log-rank *p* = 0.013) and 2-year rate of ischemic stroke of 10.5% (*p* = 0.023). Out of the 5 ischemic strokes recorded in the medical treatment arm, 4 occurred in patients with atrial septal aneurysm or hypermobility. The intervention proved relatively safe with one patient developing pseudoaneurysm at the puncture site, one suffering from pericardial effusion and 2 patients developing AF. Follow-up MRI at a median of 6 months revealed a non-significant trend of increased incidence of silent brain infarction in the medical treatment group [8.8% [3/34] in the PFO closure group vs. 18.4% [7/38] in the medication-only group, *p* = 0.24]. According to these results, 10 patients with cryptogenic stroke and PFO with high-risk features would need to be treated to avoid one stroke in 2 years.

## Meta-Analysis

An overview of the RCTs is shown in Table 2. A methodological robust systematic review and meta-analysis of the six trials with inclusion of unpublished data from the CLOSE trial, reported a total of 37 recurrent ischemic strokes occurring among 1,889 patients randomized to PFO closure, compared to 79 strokes among 1,671 patients randomized to antithrombotic therapy (pooled RR 0.36, 95% CI, 0.17–0.79, *P* = 0.01), corresponding to a number needed to treat of 131 to prevent 1 recurrent stroke during 1 person-year of follow-up (35). Turc et al. (35) concluded that despite this moderate absolute risk reduction, the benefit could be clinically significant in the long-term despite the paucity of data regarding the very long-term risk (>10 years) of stroke recurrence in younger (<60-yr-old) patients with PFO-associated “cryptogenic” stroke. RESPECT and CLOSE trials achieved follow-up of at least 5 years in more than 50% of patients and the Kaplan–Meier curve of the antithrombotic therapy group did not suggest a decline in the rate of recurrent stroke over time. Risk reduction was more pronounced in patients with high-risk PFO (atrial septal aneurysm or large shunt). In patients with higher-risk anatomical features, the pooled RR for PFO closure was 0.27 (*P* = 0.01), whereas there was a moderate non-significant trend for RR at 0.80 (*P* = 0.41) in patients with lower-risk anatomical features. An overview of echocardiographic characteristics in patients included is shown in Table 3. New-onset AF has been recorded in 93 patients randomized to the PFO closure group, 5 of whom experienced recurrent stroke. AF was transient in 66%patients. The overall rate of major procedural complications remained low at 2.4%, none of which led to death. Although this result might be explained by a lower prevalence of medical comorbidities and an upper age limit of 60 years in most trials, there was considerable heterogeneity across studies (*I*^2^ = 77%). A network meta-analysis comparing the different PFO closure devices concluded that both AMPLATZER and Gore REDUCE PFO occluders had comparable efficacy in terms of recurrent stroke reduction and were superior to the Starflex device (25).

**Table 2:** Overview of PFO closure randomized-controlled clinical trials.

**Trial name (Year)**	**PFO Device used and medical therapy**	**Control arm (s)**	**N**	**Mean age (years)**	**Follow-up (years)**	**Cross-over to closure in “control” arms**	**Primary endpoint**	**Closure group**	**Control group**	**Conclusions**
CLOSURE I (24) (2012)	STARFlex device	Asp and/or warfarin (INR 2–3)	909	46	2	–	Composite of stroke/ TIA, all-cause mortality, death from neurological causes	5.5%	6.8%	Closure not superior to medical therapy
PC Trial (26) (2013)	Amplatzer PFO Occluder	Antiplatelet therapy or oral anticoagulation	414	44.5	4.1	28 (13%)	Composite of death, non-fatal stroke, TIA, or peripheral embolism	3.4%	5.2%	Closure not superior to medical therapy
RESPECT (29) (2017)	Amplatzer PFO Occluder	Asp or Warfarin or Clop, or Asp with dipyridamole	980	45.9	5.9	19 (4%)	Composite of recurrent non-fatal ischemic stroke, fatal ischemic stroke, or early death after randomization	ITT 0.58 events per 100 patients/year. New stroke of unknown mechanism 0.31 events per 100 patients/year	ITT 1.07 events per 100 patients/year. New stroke of Unknown mechanism 0.86 events per 100 patients/year	Closure superior to medical therapy on extended follow-up in intention-to-treat analysis
CLOSE (30) (2017)	Any CE marked PFO device	Antiplatelet arm: Asp or Clop or Asp with dipyridamole-Oral anticoagulant arm: VKA or NOACs	663	43.7	5.3	3 (1%)	Recurrent fatal or non-fatal stroke	Closure vs. antiplatelet therapy: 0	Closure vs antiplatelet. Therapy 4.9% 5-year Estimate Anticoagulant vs. Antiplatelet therapy 1.5 vs. 3.8%, 5-year estimate	Closure superior to antiplatelet therapy in the presence of atrial septal aneurysm or large shunt. Anticoagulation equivalent to Antiplatelet therapy
REDUCE (33) (2017)	Helex Septal Occluder and Cardioform Septal Occluder	Asp or clop or asp with dipyridamole	664	45.2	3.2	14 (6%)	(1) Recurrent stroke (2) New brain infarct inclusive of silent brain infarct	Ischemic stroke: 1.4%. New brain infarct:5.7%	Ischemic stroke:5.4%. New brain infarct:11.3%	Closure superior to antiplatelet therapy
DEFENSE-PFO (34) (2018)	Amplatzer PFO Occluder	Asp or asp and clop, or asp and cilostazol, or warfarin	120	51.8	2.8	4 (7%)	Stroke, vascular death or TIMI defined major bleeding	Ischemic stroke: 0 2-year event rate: 0	Ischemic stroke: 10.5% 2-year event rate: 12.9%	Closure in the presence of “high risk” PFO characteristics lower rate of ischemic stroke vs. medical therapy

*ITT, Intention to treat; Asp, aspirine; Clop, clopidogrel; AVK, vitamin K antagonists; NOACs, novel anticoagulants*.

**Table 3:** Echocardiographic characteristics in patients included in the four positive RCTs.

**Trial**	**Patient selection**	**Right-to-left shunt and ASA presence**
RESPECT 2017 (29)	Unselected PFO	Grade I (1–9 MB) 22% Grade II (10–20 MB) 26% Grade III (>20 MB) 50% ASA 36%
CLOSE (30)	High risk PFO or PFO+ASA	Large (>30 MB) ASA 34%: base of aneurysm ≥ 15 mm and excursion > 10 mm
Gore REDUCE (33)	Unselected PFO	Small (1–5 MB) 19% Moderate (6–25 MB) 40% Large (>25 MB) 41% ASA 20%
DEFENSE-PFO (34)	High risk PFO or PFO+ASA	PFO size ≥2 mm (maximum separation of the septum primum from the secundum) ASA 8%: Hypermobility (phasic septal excursion into either atrium ≥10 mm)

*MB, microbubbles*.

## Patient Selection for PFO Closure

PFO is common among younger patients with cryptogenic stroke. It has been estimated that there might be 345,000 patients aged 18–60 with cryptogenic stroke harboring a PFO worldwide (36). Only a fraction will benefit from PFO closure and it is crucial that interventions be reserved for the subgroup of these patients that have a high-risk PFO. Extensive diagnostic work-up is crucial to exclude other causes of stroke.

### Ruling Out Atherosclerotic Stroke

According to the TOAST classification, an ischemic stroke event is attributed to large-artery atheromatosis (LAA) when the culprit lesion produces a stenosis >50%. It has been suggested that luminal narrowing is not a prerequisite for plaque vulnerability (37), and it is known that most coronary artery plaques resulting in myocardial infarction are associated with <50% luminal stenosis (38). There have been many reports that challenged the strict TOAST criteria for LAA stroke; in the Causative Classification System (CCS) of acute ischemic stroke, besides degree of stenosis, plaque characteristics as well as history of multiple TIAs/strokes and pattern of ischemic lesions in MRI are taken into consideration before ruling out the possibility that atherosclerosis had a causative relation to the ischemic stroke (39). In the Subtypes of Ischaemic Stroke Classification System (SPARKLE), using a total plaque area cutoff ≥1.19 cm^2^ resulted in the classification of 33% of cases as LAA-related strokes compared to only 21% based on TOAST criteria (40). There are most likely atheromatous plaques resulting in <50% stenosis and provoking LAA stroke/TIA through distal embolism, which, in the absence of other definite cause of stroke, might be classified as PFO-related, but in fact these represent missed LAA strokes. In a seminal study of black-blood MRI examination of the carotid arteries in patients with cryptogenic hemispheric stroke and atherosclerotic plaques thicker than 2 mm in the carotid bifurcations, AHA type VI plaques [complicated plaques with intraplaque hemorrhage, thrombus, or rupture of the fibrous cap, (41) known to be overrepresented in symptomatic stenotic ICA plaques (42)] were found exclusively on the ipsilateral side of stroke (37.5 vs. 0.0%) (43). The results were replicated using a routine MR angiography protocol performed on the day of stroke; intraplaque high-intensity signal was once again exclusively found in non-stenotic plaques ipsilateral to the cryptogenic cerebral infarction (22 vs. 0%). Non-stenotic plaques ≥5 mm thick on CT angiography were present in 11% of ipsilateral and 1% of contralateral arteries in patients with Embolic Stroke of Undetermined Source (ESUS); plaques ≥4 mm thick were present in 19 and 5%, respectively and plaques ≥3 mm thick were present in 35 and 15%, respectively (44). This important difference of 20% could correspond to up to one in five ESUS patients having non-stenotic LAA stroke that would not benefit from PFO closure in case they also harbor a PFO, but would most certainly benefit from strict cardiovascular risk factor modification, aggressive short-term antiplatelet therapy and high-intensity statin treatment. In line with these findings, a recent study has shown close interrelation between increased arterial stiffness, aortic atheroma, reduced endothelial glycocalyx thickness and presence of cryptogenic stroke (45). High-risk intracranial plaques with mild degree or even no stenosis (thus impossible to discover with lumen imaging) are more prevalent than previously acknowledged and are associated with ischemic stroke and unfavorable outcomes (46). High-resolution vessel wall MRI could identify the high-risk plaque features of intracranial arteries and may thus serve as a promising tool to exclude these patients from unnecessary PFO closure. A most recent article proposes a working definition of symptomatic non-stenotic carotid disease (SyNC) to further promote research in the role of SyNC in stroke etiology (47).

### Ruling Out AF-Related Stroke

Up to 30% of patients with cryptogenic stroke have shown episodes of paroxysmal AF detected with inserted loop recorders (ILRs) in the following 3 years after stroke (48). However, AF is mostly a disease of older age and rates of AF detected in the PFO closure trials were low in the control arms, as patients in most trials were under 60 years old. In the CRYSTAL-AF trial (Cryptogenic Stroke and Underlying AF), 3 years of continuous monitoring with an ILR revealed AF in only 3% of cryptogenic ischemic stroke patients aged <54 years and in 4% aged between 54 and 61 years (49). In younger stroke patients continuous monitoring is unlikely to detect AF but as patients' age approaches 60 years, it might be necessary to perform cardiac rhythm monitoring for more than 24 h, ideally for at least a week. More prolonged ambulatory cardiac monitoring is reasonable in patients with structural, electrophysiological, or blood biomarker-related evidence of atrial cardiopathy (50). Low stroke recurrence rates in PFO-associated stroke under medical treatment might allow for several months of post-ponement of PFO closure before excluding paroxysmal AF beyond reasonable doubt. ILR revealed an occult AF in more than a third of cryptogenic stroke patients that were considered candidates for PFO closure in an Italian single-center series; even if patients have been recruited for a decade, meaning most of the recruitment took place before the recent positive RCTs that clarified PFO closure eligibility criteria, it highlights the importance of ILR for AF detection in cryptogenic stroke patients (51). In our center, we routinely propose ILR placement according to the flow chart for AF screening of a European position paper on the management of patients with PFO (52): patients <55 years may be considered for ILR when they have high clinical suspicion of AF (i.e., ≥2 high-risk factors for AF: congestive heart failure, uncontrolled hypertension, uncontrolled diabetes mellitus, structural heart alterations); for those over 55 years of age the presence of any major risk factor for AF is sufficient to prompt ILR insertion. Echocardiographic findings such as left atrial dilation or frequent atrial runs in Holter electrocardiograms should also prompt for ILR placement. Finally, increased arterial stiffness has been linked with impaired left atrial strain in patients with cryptogenic stroke implying the presence of conditions that facilitate atrial myopathy, atrial fibrillation and thus cardioembolism. In this study 18% of the patients with ESUS had evidence of PFO (45).

### Ruling Out Small Vessel Disease

Up to a quarter of strokes are due to cerebral small vessel disease (SVD). Milder symptoms and better prognosis compared to other ischemic stroke types, complex and multifactorial pathogenesis, and increased risk of cerebral hemorrhage, are some reasons behind the absence of novel interventions to reduce recurrence in SVD stroke (53). Facing a small subcortical ischemic lesion in a stroke patient without other obvious cause of stroke, the clinician has to depend on neuroimaging characteristics of SVD (leukoencephalopathy, microbleeds, older lacunes) and patient history (hypertension, diabetes mellitus especially if other manifestation of systemic SVD such as microalbuminuria are present) to reach a diagnosis of acute lacunar stroke.

### The RoPE Score

It is therefore crucial to set apart stroke patients with either atherosclerosis or SVD harboring an incidental PFO as these patients will be unnecessarily submitted to intervention. Multivariate modeling in 12 PFO studies in cryptogenic stroke, revealed many factors influencing the odds of PFO detection. The Risk of Paradoxical Embolism (RoPE) Point Score has been developed based on the similarity of the odds ratios in this model, as an index to stratify cryptogenic stroke patients with PFO by their likelihood that the stroke was related to their PFO (19). Absence of history of hypertension, diabetes, other TIA/stroke, smoking or presence of cortical infarct on imaging, contribute one point each. Age is of outmost importance, counting for 5 points in very young patients (aged 18–29) and for 0 points for those over 70. High RoPE scores correspond to a high PFO-attributable fraction, eg a RoPE score of 9 is associated with a 88% chance that the PFO found in a cryptogenic stroke patient is the cause of stroke. This score appears to be a promising tool to argue in favor of non-PFO-associated causes of stroke in patients that do not fulfill strict criteria of LAA or SVD-related stroke. Further analysis of RoPE database revealed that patients with low RoPE scores (indicating that the index event was less likely to be PFO-related) experience recurrent strokes in relation to conventional vascular risk factors such as age, whereas the echocardiographic PFO characteristics do not seem to affect the recurrence risk. On the other hand, the echocardiographic characteristics of PFOs in those with high RoPE scores are strongly associated with recurrence, suggesting different stroke mechanisms for these different strata (54). Interestingly, the CLOSE trial reported a high RoPE score of 7.4. Finally, a Portuguese study recently reported that ROPE scores ≤ 6 independently predicted higher risk of recurrent stroke and mortality in cryptogenic stroke patients who underwent PFO closure. This observation provides further insight that low ROPE scores may assist in identifying cryptogenic stroke patients with incidental and not causally related PFOs (55).

### Paradoxical Embolism

We still do not know how PFO sends emboli to the brain. In only rare instances is a patient with cryptogenic and potentially PFO-associated stroke found to suffer from concomitant deep vein thrombosis (DVT), suggesting, if not proving, paradoxical embolism. These patients may be protected from subsequent paradoxical brain embolism with PFO closure but remain at risk of subsequent pulmonary embolism without proper management and anticoagulation treatment. One large multicenter prospective study, the Paradoxical Emboli From Large Veins in Ischemic Stroke (PELVIS) study, found an 11.6% prevalence of pelvic DVT in ischemic stroke patients, with increased frequency in cryptogenic stroke cases, suggesting that there could be patients with DVT missed by lower limb ultrasound (56). However, a large single-center study on the utility of deep pelvic venous imaging for the diagnostic evaluation of paradoxical embolic source in cryptogenic stroke or TIA patients with PFO, found a 10-fold lower prevalence of pelvic DVT (2.1 vs. 20.0%) (57). These results may have discouraged further research and we are not aware of any new study on the subject since the publication of the aforementioned study in 2014. It should be noted that patients with proven DVT around the time of stroke onset may require life-long anticoagulation instead of PFO closure. This has been suggested by the RESPECT trial that reported higher rates of DVT and pulmonary embolism in PFO-treated patients (29). In fact, both treatment arms showed higher rates of DVT/PE than the healthy population. Further analysis showed that among patients in the PFO closure group, the propensity to venous thromboembolic events was particularly high in the subgroup of patients who had previous unprovoked DVT. Despite representing only 4% of patients in the PFO closure group, they accounted for 25% of the venous thromboembolic events that occurred during the trial. This is a subgroup of PFO stroke patients that require anticoagulation treatment even after PFO closure and some experts argue that there is no benefit of intervention on top of anticoagulation treatment, as proposed in current clinical guidelines.

## International Guidelines

Many national and international guidelines have been adapted to the positive findings of multiple RCTs. All guidelines are in favor of PFO closure in eligible patients and agree that proper patient selection is key to patient benefit. Minor differences involve the role of anticoagulation in medically treated patients, intervention for those over 60 and individual patient characteristics that may strengthen or alleviate causality of PFO. A group of European medical associations has provided a comprehensive position paper on the management of patients with PFO (52). The first step in the algorithm proposed is to establish a high possibility of causal link between a cryptogenic TIA/stroke and PFO presence. Multidisciplinary teams should consider clinical data, neuroimaging studies, echocardiographic findings and if no other cause is found, intervention should be proposed to patients 18–65 years old after discussing risks and benefits. The Heart and Stroke Foundation Canadian Stroke Best Practice Committees propose similar guidelines, restricting patient age to 18–60 years, consistent with most RCTs. For patients requiring anticoagulation for other indication, guidelines do not propose PFO closure, as benefit may be less important (58). A United Kingdom expert panel has provided a strong recommendation in favor of PFO closure plus antiplatelet therapy compared with antiplatelet therapy alone, a weak recommendation in favor of PFO closure plus antiplatelet therapy compared with anticoagulants and a weak recommendation in favor of anticoagulants compared with antiplatelet therapy (59).

## Critical Appraisal of Reduction of Disability and Cost-Effectiveness of the Intervention

The most problematic part of assessing the recent results of the RCTs results are so far missing patient-oriented outcome evaluations and benefits are not weighted against procedural risks (60). Both observational studies and RCTs agree that there is a very low risk of recurrent stroke under medical treatment, around 1% annually, leaving little margin for complications. What is even more important is that PFO associated stroke is usually non-disabling. Data from the RESPECT trial indicate that the reduction in recurrent strokes with PFO closure (16 strokes in the medical management group vs. 6 in the closure group) was due to a reduction in non-disabling strokes; disabling strokes (modified Rankin Scale score 3 or greater) were 4 in each treatment arm (61). In CLOSE trial there were 14 major device- and/or procedure-related complications that exactly equaled the number of recurrent strokes in the antiplatelet-only group (see Table 2). In the same study, only 1 out of 14 strokes was disabling [modified Rankin scale score (mRS) of 3 or higher]. As already discussed, CLOSE showed clearly positive results in favor of PFO closure but, in the light of the observation that strokes prevented are mostly mild strokes, it remains unclear why complications should be weighted less than efficacy endpoints. The issue of PFO-associated strokes being mostly clinically minor raises doubts about the efficacy of PFO closure to reduce disability and, consequently, the cost-effectiveness of the procedure (62). The CLOSE data indicate a number needed to treat that is >100 to prevent 1 stroke leading to any functional disability (mRS≥2) and >200 to prevent 1 disabling stroke (mRS≥3) within the subsequent 5.2 years, values much more higher that the usually presented on prevention of all strokes. This is an important parameter in order to assess cost-effectiveness of the procedure, since lower disability means lower long-term cost In a cost effectiveness study (63) annual post-hospitalization costs based on mRS were taken from a previously published stroke model based on a cohort of 958 acute stroke patients in US (64) and utility values were taken from previously validated stroke models using the same health states (65). Both stroke models involved large-vessel-occlusion (LVO) strokes. In the latter study, the probability of being independent, dependent, or deceased in each treatment arm was calculated using the data provided from 5 RCTs of mechanical thrombectomy for LVO stroke. LVO strokes have very high disability and mortality rates, representing just a third of ischemic strokes but being responsible for three-fifths of dependency and more than nine-tenths of mortality after ischemic stroke (66). This is in sharp contrast with PFO-associated strokes that are usually mild and they should therefore not be used in PFO-associated stroke models.

## Real-World Data

As far as safety of the procedure is concerned, real life registries have repeatedly shown significant procedure-related complication rates that exceed those of RCTs. In a retrospective cohort study, total adverse outcomes were observed at the rate of 7%, mostly new onset of AF, (67) much greater than the 1% reported in RCTs (Table 4). The Society for Cardiovascular Angiography and Interventions (SCAI) has proposed standards of procedural and operator requirements for the establishment and maintenance of PFO closure programs (68). The procedure should be performed by an experienced physician, in a high-volume center on structural/congenital cardiac catheterizations that maintains a minimum number of septal interventions per year. Multidisciplinary teams should be organized to properly select and manage patients with cryptogenic PFO-attributable stroke. A period of extended cardiac monitoring should be performed for approximately 4 weeks in patients over the age of 40. As mandated by the FDA, patient selection should involve close collaboration between the PFO proceduralist and a neurologist (preferably a stroke neurologist) (69). Inclusion and exclusion criteria used in the RCTs should guide center-specific predetermined algorithms for selecting patients. It is of outmost importance the continuous recording of data on all cases with quality assessment and quality improvement process. Data should include patient characteristics, indication for the procedure, procedure performance, and up to 30-day outcomes. Independent measurement of outcomes is crucial to establish the safety of the procedure in real world clinical settings. Although participation in a registry is currently not mandatory for the use of the currently approved devices, the management of PFO-associated stroke is a field of continuous research and there are many questions that have not yet been answered. We need high-quality real-world data and submission of all cases to a national or multicenter registry, for benchmarking is the safest way to confirm the external validity of the promising results of RCTs.

**Table 4:** Device and procedure-related adverse events in the four positive RCTs among the patients in the PFO Closure Group.

Gore REDUCE (33) 441 patients	At least one serious procedure-related adverse event in 11 patients (2.5%): Serious bleeding 4, device dislocation 2, hypotension 2, anxiety, aortic dissection, arteriovenous fistula, cardiac tamponade, chest discomfort, complication of device removal, fatigue, hemiparesis, incision site hematoma, incision site hemorrhage, non-cardiac chest pain, post-procedural hemorrhage, puncture site hemorrhage, respiratory arrest. Serious device-related adverse event in 6 patients (1.4%): Device dislocation 3, device-related thrombosis 2, aortic dissection
RESPECT 2017 (29) 499 Patients	Serious Adverse Events Related to the Procedure or Device in 21 patients (4.2%): Atrial fibrillation 2, ischemic stroke 2, cardiac thrombus 2, pericardial tamponade 2, pulmonary embolism 2, residual shunt requiring closure 2, bleeding 2, atrial flutter, cardiac perforation, chest tightness, deep-vein thrombosis, infective endocarditis, pericardial effusion, sepsis, non-sustained ventricular tachycardia, allergic drug reaction, hematoma, vasovagal reaction
CLOSE (30) 238 patients	Major or fatal device-related or procedure-related complication in 14 patients (5.9%): Atrial fibrillation 9, supraventricular tachycardia 2, atrial flutter, air embolism, and hyperthermia resulting in prolongation of hospitalization
DEFENSE PFO (34) 60 patients	Major procedural complications occurred in 2 patients (3.3%): Pericardial effusion, pseudoaneurysm at the puncture site. Atrial fibrillation developed in 1 patient 1 day after the procedure and in another patient during follow-up

## Future Directions

Many areas regarding PFO closure will be hopefully elucidated in the following years (70). Further research is required to clarify the cause-effect relationship between PFO and cerebral embolism in order to advance patient selection and improve secondary prevention. Post-approval prospective studies like the International PFO Consortium and AMPLATZER PFO Occluder Post-Approval Study (PFO PAS) are currently recruiting patients and will eventually further elucidate risks and benefits of the procedure^1^^,^^2^. In the following years we will see more devices entering clinical practice; the PROOF Trial is a prospective, open-label study on Percutaneous PFO Closure Using the Occlutech PFO Occluder^3^. The role of anticoagulation has not been decidedly refuted in PFO-associated stroke; on the contrary, data from a pooled analysis of CLOSE and subgroups of patients with a PFO in two trials comparing oral anticoagulants to antiplatelets in patients with cryptogenic stroke, suggest that anticoagulants may be superior to aspirin, with a 52% reduction in stroke recurrence (odds ratio 0.48, *P* = 0.04; no heterogeneity was identified) (71). A recent network metanalysis has similar results (71 fewer ischemic strokes per 1,000 patients treated with anticoagulation) (72). We do not know whether patients who were excluded from RCTs, particularly those >60 can benefit from PFO closure (73); only a handful of patients of this age group were included in the DEFENSE PFO trial. DEFENSE-ELDERLY is a currently recruiting RCT that aims to evaluate the prevalence and clinical impact of AF in elderly ESUS patients and no other known sources of stroke besides a high-risk PFO, and compare it with elderly ESUS patients without high-risk PFO^4^. Novel PFO closure devices may improve closure rates and decrease closure complications. It is possible that life-long antiplatelet treatment is not needed in all patients post PFO closure (74). Last but not least, cost-effectiveness of PFO closure remains to be proven after accumulating robust data on clinical outcome after PFO-associated stroke.

## Conclusions-Take Home Messages

PFO represents a potential source of cerebral embolism in subgroup of cryptogenic stroke patients and its mere presence in patients with ischemic stroke is insufficient to prove causality. Multiple recent RCTs have established the role of PFO closure in cryptogenic stroke patients 60 years old or younger. Since PFO is commonly identified in cryptogenic stroke patients, it is crucial to select patients that will gain most benefit from the intervention. PFO-related stroke is a diagnosis of exclusion that requires a comprehensive work-up that fails to provide an alternative etiology. A multidisciplinary team of an expert stroke clinician and a cardiologist may integrate clinical, neuroimaging and echocardiographic findings in order to detect the appropriate subgroup among cryptogenic stroke patients that may benefit from intervention. A combination of high RoPE score and echocardiographic features described in the recent positive RCTs may assist experienced vascular neurologists in properly selecting cryptogenic stroke patients with high-risk PFOs for PFO closure by experienced interventional cardiologists with close surveillance for efficacy and safety outcomes. In our centers, we insist in a full diagnostic work-up tailored to individual patient characteristics: any sign for atherosclerosis will prompt for a high-resolution MRI of intracranial and extracranial vessels in search of non-stenosing but active plaques; any hint for occult AF will prompt for ILR placement. Further data on PFO-associated stroke pathogenesis, real-world data on PFO closure and data on clinical outcome of PFO-associated stroke are urgently needed.

## Author Contributions

AS wrote the first draft of the document. KP, OK, II, MD, and KT wrote sections of the manuscript. PK provided figures and critical revision. GT final revision. All authors contributed to manuscript revision, read, and approved the submitted version.

## Conflict of Interest

The authors declare that the research was conducted in the absence of any commercial or financial relationships that could be construed as a potential conflict of interest.

## Footnotes

^1^ClinicalTrials.gov Identifier: NCT00859885

^2^ClinicalTrials.gov Identifier: NCT03309332

^3^ClinicalTrials.gov Identifier: NCT04029233

^4^ClinicalTrials.gov Identifier: NCT04285918

## Supplementary Material

Supplementary Video 1 PFO detection with agitated saline contrast during Valsalva maneuver. 2D TEE demonstrates microbubbles crossing the PFO through a septal separation.

Supplementary Video 2 3D TEE showing septal separation.
